# Immunomodulatory properties of cacao extracts – potential consequences for medical applications

**DOI:** 10.3389/fphar.2013.00154

**Published:** 2013-12-12

**Authors:** Kathrin Becker, Simon Geisler, Florian Ueberall, Dietmar Fuchs, Johanna M. Gostner

**Affiliations:** ^1^Division of Biological Chemistry, Biocenter, Innsbruck Medical UniversityInnsbruck, Austria; ^2^Division of Medical Biochemistry, Biocenter, Innsbruck Medical UniversityInnsbruck, Austria

**Keywords:** cacao, cocoa, anti-inflammatory, immunology, neurobiochemistry, tryptophan metabolism

## Abstract

Anti-inflammatory properties of cacao, fruits of *Theobroma cacao* L. (Sterculiaceae), are well documented, and therapeutic applications are described for gastrointestinal, nervous, and cardiovascular abnormalities. Most, if not all of these disease conditions involve inflammation or immune activation processes. The pro-inflammatory cytokine interferon-γ (IFN-γ) and related biochemical pathways like tryptophan breakdown by indoleamine 2,3-dioxygenase (IDO) and neopterin formation are deeply involved in their pathogenesis. Neopterin concentrations and the kynurenine to tryptophan ratio (Kyn/Trp, an estimate of IDO activity) are elevated in a significant proportion of patients with virus infections, cancer, autoimmune syndrome, neurodegeneration, and coronary artery disease. Moreover, higher neopterin and Kyn/Trp concentrations are indicative for poor prognosis. When investigating the effect of aqueous or ethanolic extracts of cacao on IFN-γ, neopterin and Kyn/Trp concentrations in mitogen-stimulated human peripheral blood mononuclear cells, breakdown of tryptophan by IDO, and formation of neopterin and IFN-γ were dose-dependently suppressed. The effects observed in the cell-based assays are associated with the antioxidant activity of the cacao extracts as determined by the cell-free oxygen radical absorption capacity assay. The influence of cacao extracts on IDO activity could be of particular relevance for some of the beneficial health effects ascribed to cacao: tryptophan breakdown by IDO is strongly involved in immunoregulation, and the diminished availability of tryptophan limits the biosynthesis of neurotransmitter serotonin. The inhibition of tryptophan breakdown by cacao constituents could thus be relevant not only for immune system restoration in patients, but also contribute to mood elevation and thereby improve quality of life. However, the available data thus far are merely *in vitro* only and future studies need to investigate the influence of cacao on tryptophan metabolism *in vivo*.

## INTRODUCTION

Epidemiological studies suggest that high dietary intake of secondary plant metabolites is associated with a decreased risk of diseases like cardiovascular disorders or cancer (Surh, 2003). Tea, coffee and cocoa, fruits and vegetable are rich in polyphenols, a complex group of substances that gained considerable interested due to their antioxidative properties, thus supporting potential beneficial implications on human health. Meanwhile, more than 8000 different phenolic structures have been identified (Bravo, 1998).

Cocoa is the term for unroasted fruits (beans) of the evergreen cacao tree *Theobroma cacao* L. (Sterculiaceae or alternatively Malvaceae). After grinding of the cocoa seeds, the cocoa butter is removed from the dark, bitter cocoa solids. The consumption of cocoa can be dated back to the preclassic Maya as early as 600 B.C. (Hurst et al., 2002). Drinking of beverages prepared with cocoa for therapeutic purpose was very popular in Olmec, Maya, and Aztec cultures. According to tradition, liquid cocoa is a divine drink, which builds up resistance and fights fatigue. “A cup of this precious drink permits a man to walk for a whole day without food” (traditional citation of Hernán Cortés, 1519; Corti et al., 2009). Until today, cocoa consumption is associated with regalement and a sense of delight. The most known applications of cocoa for medicinal purposes arise from appetite stimulating, relaxing, and mood-enhancing effects.

Also, cacao-rich chocolate has been known for centuries and although common processing strategies of cocoa beans, such as roasting or fermentation, reduce the amount of some bioactive constituents (Payne et al., 2010), dark chocolate consumption has been suggested to promote beneficial effects on human health (Engler et al., 2004), whereby the content of low molecular weight flavanols, e.g., of epicatechin, is supposed to be of particular importance.

The discovery of a wide range of biologically active substances has changed the perception of cocoa/cacao as stimulant or luxury food only, and several *in vitro* and *in vivo* studies suggest that some identified active compounds exhibit pharmacologic effects with potential health implications. A large number of studies reported on benefit exerted by cacao/cocoa extracts and constituents on processes involved in inflammation or impaired immune functions, ageing, blood pressure regulation, atherosclerosis, or cardiovascular diseases development (Galleano et al., 2009; van Dam et al., 2013; Williams, 2013). Cocoa compounds were shown to influence on, e.g., platelet activation (Rein et al., 2000) and nitric oxide (NO)-dependent activities (Fisher et al., 2003; Heiss et al., 2003) as well as on cytokine production *in vivo*. Furthermore, a beneficial effect of cocoa consumption on blood pressure (Grassi et al., 2010), insulin resistance, and vascular and platelet function has been suggested (Corti et al., 2009; Galleano et al., 2009).

Among the most discussed effects of cacao/cocoa uptake are interferences with vascular function, platelet reactivity and inflammatory processes, suggesting cardioprotective properties of some constituents (Keen et al., 2005). A variety of environmental and genetic factors may have influence on cardiovascular disease development, and several pathological changes may lead to dysfunction of pathways that are involved in the maintenance of cellular and tissue homeostasis, promoting conditions of oxidative stress (Ramsey et al., 2010). The “oxidative modification hypothesis” describes an important process in atherosclerosis disease development, where lipids and proteins of the vessel wall and low-density lipoprotein (LDL) are oxidized in an early stage of the disease. The accumulation of lipids in the arterial wall leads to chronic inflammation. It is accompanied by thickening and hardening of the vessel wall, which lowers elasticity and results in impaired blood flow (Brigelius-Flohé et al., 2005). However, pathological changes occur long before diseases are diagnosed, making it difficult to treat real primary causes rather than symptoms.

Among the major mechanisms that contribute to the potential cardioprotective effects of cocoa flavanols are their direct and indirect antioxidative properties. The high dietary intake of antioxidant compounds is suggested to decrease the atherosclerotic risk by attenuating oxidation processes (Brigelius-Flohé et al., 2005) and/or by interfering with cellular signaling pathways (Schroecksnadel et al., 2007; Stevenson and Hurst, 2007). Still, the correlation between antioxidant intake and reduction of cardiovascular disease is challenging (Joshipura et al., 2001; Galleano et al., 2009) and also, the prevention of LDL oxidation by antioxidants is controversially discussed in literature (van de Vijver et al., 1997; Brigelius-Flohé et al., 2005).

However, the inflammatory background in disease development, like the starting point of pro-inflammatory cytokine release and metabolite production, is a central target for preventive actions. In line with this, we aim to discuss potential interferences of cacao/cocoa antioxidants with central immunoregulatory mechanisms, by focusing on pathways involved in cell-mediated immune response.

## ANTIOXIDANT CONTENT OF COCOA/CACAO

Antioxidants are reducing chemicals that are able to inhibit or prevent oxidation processes of molecules by being oxidized themselves. An antioxidant can also be a radical scavenger, which can transform itself into a rather inert radical, and terminates radical-driven chain reactions (Brigelius-Flohé et al., 2005). Antioxidant compounds may be produced within the human body or can be absorbed from dietary intake. Most plants and herbs are rich sources of antioxidant molecules and numerous epidemiological studies were made investigating the potential radical scavenging potential of plant-derived phenols and polyphenols *in vivo* (Albarracin et al., 2012).

Cocoa/cacao contains remarkable amounts of total phenolics and flavonoids in comparison to other fruits and vegetables. The phenolic content of roasted cocoa beans can account for up to 18% of total weight, with catechins and flavan-3-ols, anthocyanins, and proanthocyanidins as major groups (Rusconi and Conti, 2010). In particular epicatechin, catechin, gallocatechin, and epigallocatechin are contained in high concentrations. The flavanol content (catechin and epicatechin content) of chocolate is 460–610 mg/kg and thus comparable to that in green tea and beans (Corti et al., 2009). In summary, over 380 compounds of cocoa have been identified, including theobromine and caffeine (at low levels), as well as 10 psychoactive compounds (Rusconi and Conti, 2010).

Both, the flavanol content and the total antioxidant capacity increases in plasma after oral intake of cocoa or dark chocolate (Serafini et al., 2003; Spadafranca et al., 2010). Consumption of cacao in combination with milk or milk chocolate was shown to reduce these effects dramatically, although such matrix-dependent absorption effects are still matter of discussion (Corti et al., 2009).

Miller et al. (2006), compared cacao and popular chocolate products with the oxygen radical absorbance capacity test (ORAC) and determined polyphenol and procyanidin contents. Natural cocoa powders contained the highest levels of antioxidant capacity (720 and 875 μmol Trolox equivalents/g) as well as higher total polyphenols and procyanidins, compared to other products (Miller et al., 2006). Of course, food processing such as roasting, fermentation, or conventional chocolate manufacturing leads to a decrease of antioxidant content (Corti et al., 2009; Payne et al., 2010).

Several antioxidants have also the capacity to influence cellular signaling pathways and thus activate the expression of protective, antioxidant or immunoregulatory genes. Experiments of Rodríguez-Ramiro et al. (2011) demonstrated that cocoa phenolic extracts can improve the redox status of acrylamide-treated Caco-2 intestinal carcinoma cells by inhibiting glutathione consumption and reactive oxygen species (ROS) generation, by increasing the levels of gamma-glutamylcysteine synthetase and glutathione-*S*-transferase and by blocking of apoptotic pathways.

It can be suggested that, e.g., the reduction of lipid and inflammatory biomarkers in hypertension by some cocoa-containing products (Solà et al., 2012) is a concerted action of both, direct and indirect antioxidant properties of cocoa/cacao.

## INTERFERENCES OF COCOA/CACAO EXTRACTS WITH CELL-MEDIATED IMMUNE RESPONSE

Various immunocompetent cells and mediators are involved in the regulation and modulation of immunological reactions, and especially the interaction of lymphocytes and macrophages plays an important role (Balakrishnan and Adams, 1995). Different types of immune responses are completed by distinct subsets of T-helper (Th) cells like Th1-, Th2-, and Th17-type cells (Romagnani, 2006; Jin et al., 2012). During Th1-type (=cell-mediated) immune response, the pro-inflammatory cytokines interferon-γ (IFN-γ), and interleukin-2 (IL-2) are produced by activated T and natural killer cells to mount an effective response against pathogens and tumor cells, while allergic Th2-type cell responses are characterized by cytokines IL-4, IL-5, and IL-13 (Barth et al., 2003; Romagnani, 2004). In a kind of cross-regulation, Th1-type cytokines can suppress the Th2-type immune response and *vice versa* (Lucey et al., 1996).

Interferon-γ plays a prominent role in host defense and stimulates cellular responses, such as production of high amount of ROS by macrophages (Nathan, 1986). Toxic ROS products, as hydrogen peroxide (H_2_O_2_), hypochlorite (OCl^-^), and superoxide anion radical ($O_{2}^{-}$), suppress growth of target cells and pathogens. In inflamed tissue, increased formation of ROS leads to the disruption of protective cellular antioxidant mechanisms, which results in a milieu also called oxidative stress (Bowler and Crapo, 2002). Further, mitogen-activated protein kinase (MAPK), transcription factor nuclear factor-κB (NF-κB), and activator protein (AP)-1-dependent signaling cascades become activated and modulate the expression of pro-inflammatory cytokines, such as tumor necrosis factor-α (TNF-α) and IL-1, chemokines, and adhesion molecules (Aggarwal, 2004). NF-κB is highly inducible by oxidants and a key molecule of the pro-inflammatory signaling cascade that regulates the transcription of cytokines, chemokines, growth factors, adhesion molecules, immunoreceptors, and acute phase proteins (Asehnoune et al., 2004).

These redox-sensitive pathways regulate initiation, execution, and resolution of the inflammatory response. Thus, interferences of dietary antioxidants with ROS signaling in inflamed tissues might protect against cellular damage (Schroecksnadel et al., 2007). However, antioxidants, when supplemented in high dose, can also be unfavorable and shift Th1-type immune response toward Th2-type, promoting allergic diseases (Zaknun et al., 2012).

In several *in vitro* studies, cacao extracts were shown to down-regulate pro-inflammatory cytokines and their downstream biochemical pathways (Corti et al., 2009; Galleano et al., 2009). Ramiro et al. (2005) reported that cocoa extracts and epicatechin decreased the expression of TNF-α, monocyte chemoattractant protein 1 (MCP-1) and other cytokines, as well as NO release in macrophages. Zeng et al. (2011) reported an inhibition of $O_{2}^{-}$ production, cytokine release, and NF-κB activation after clovamide (a phenylpropenic acid amide present in cocoa) treatment in PMA (phorbol 12-myristate 13-acetate)-stimulated human monocytes. Application of phenolic extracts from unroasted and roasted cocoa beans showed similar but less strong effects.

**FIGURE 1:**
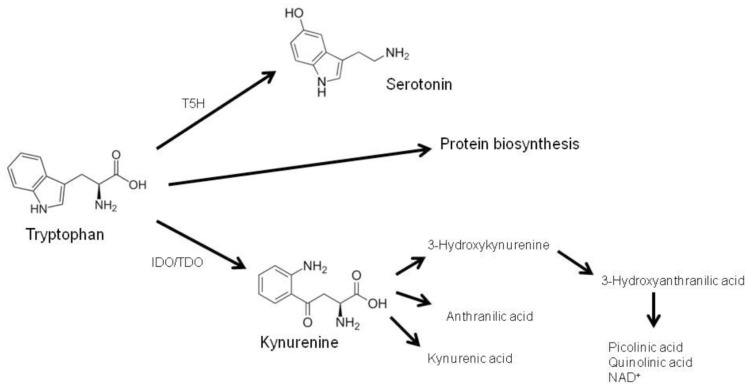
**The essential amino acid tryptophan is required for protein biosynthesis or is metabolized via two biochemical routes: (i) via tryptophan 5-hydroxylase (T5H) and subsequent decarboxylation to 5-hydroxytrytophan and serotonin, and (ii) via tryptophan 2,3-dioxygenase (TDO) and indoleamine 2,3-dioxygenase (IDO) to kynurenine, which is fur-ther converted to several metabolites (adapted from Widner et al., 2002)**.

A central immunoregulatory pathway induced by IFN-γ in human monocytes/macrophages is the breakdown of the essential amino acid tryptophan via the enzyme indoleamine 2,3-dioxygenase (IDO). Unlike the hepatic isoenzyme tryptophan 2,3-dioxygenase (TDO), IDO is expressed in a variety of cell types such as macrophages, fibroblasts, epithelial and endothelial cells, and cells of the central nervous system. IDO expression and activity is drastically increased during inflammation (Schröcksnadel et al., 2006b). About 95% of dietary tryptophan is degraded via the kynurenine pathway, leading to the formation of L-kynurenine and further downstream products kynurenic acid, anthranilic acid, 3-hydroxykynurenine, quinolinic acid, picolinic acid, and nicotinamide adenine dinucleotide (NAD^+^; Adams et al., 2012; **Figure 1**). Another metabolic route of tryptophan is the serotonin pathway, in which tryptophan is hydroxylated via the rate-limiting enzyme tryptophan 5-hydroxylase (T5H) to 5-hydroxytrytophan and serotonin (5-hydroxytryptamin, 5-HT). Therefore, tryptophan availability is strongly involved in the pathogenesis of mood disorders and depression (Widner et al., 2002), as an insufficient tryptophan concentration may cause neuropsychiatric symptoms when serotonin production is diminished (Widner et al., 2000).

The IDO-induced tryptophan breakdown is an important defense strategy to prevent undesired growth, e.g., of intracellular pathogens such as viruses, parasites, and bacteria, but also tumor cells (Pfefferkorn, 1986). In various diseases that are associated with cellular immune activation, decreased tryptophan levels together with increased kynurenine to tryptophan ratio (Kyn/Trp) ratio are found. Kyn/Trp indicates that the low amount of tryptophan results from enhanced degradation rather than from a reduced dietary intake (Fuchs et al., 1990).

**FIGURE 2:**
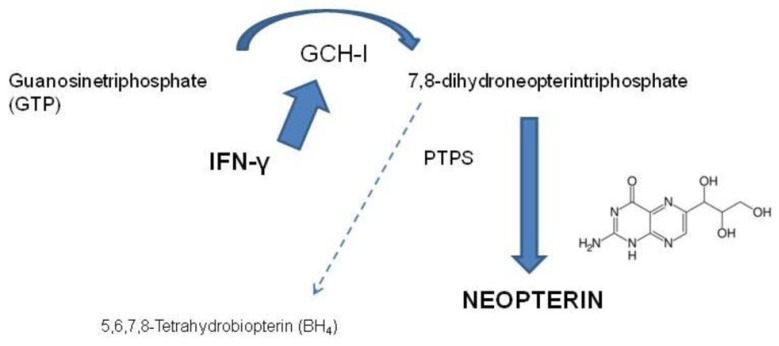
**In various cells the Th1-type cytokine interferon-γ (IFN-γ) induces the GTP-cyclohydrolase I (GCH I) to produce 7,8-dihydroneopterintriphosphate.** Due to a deficiency in pyruvoyltetrahydropterin synthase (PTPS) in human monocyte-derived macrophages and dendritic cells, the production of 5,6,7,8-tetrahydrobiopterin is almost zero and neopterin is produced in high concentrations.

In parallel to IDO, IFN-γ activates the formation of the pteridine derivative neopterin, by the enzyme guanosine triphosphate (GTP)-cyclohydrolase (GCH; Werner et al., 1989). Neopterin is a sensitive marker for activation of the immune system that has been identified in the early 1980s (Fuchs et al., 1982; Werner et al., 1987). The biosynthesis of neopterin starts from GTP, which is converted to 7,8-dihydroneopterintriphosphate by GCH. Due to a deficiency of the tetrahydrobiopterin (BH_4_)-forming enzyme 6-pyruvoyltetrahydropterin synthase (PTPS) of human macrophages and dendritic cells, the biosynthesis of BH_4_ is prevented and neopterin is produced (Werner et al., 1990; **Figure 2**). Other cell types like human endothelial cells and B-lymphocytes have been shown to be able to produce low amounts of neopterin, however, not comparable with the production of neopterin in macrophages (Andert et al., 1992; Hofmann et al., 1992).

Of note, BH_4_ is required as a cofactor of several enzymes such as T5H and inducible NO synthase (iNOS). Due to insufficient BH_4_ production, human monocyte-derived macrophages and dendritic cells are limited in the formation and release of NO and peroxynitrite (ONOO^-^; Murr et al., 2002). Some studies have shown that diseases with an inflammatory background can have endogenous NO formation and high serum neopterin levels together. This occurs probably by the stimulation of NOS in other cell types, e.g., endothelial cells (Weiss et al., 1995).

High neopterin concentrations reflect IFN-γ activity and thus can serve as a biochemical indicator of immune activation (Fuchs et al., 1992; Schroecksnadel et al., 2006a; Schröcksnadel et al., 2006b). High neopterin levels have been shown to be associated with a strong release of ROS in activated macrophages (Nathan, 1986). Accordingly, in various diseases, neopterin concentrations correlate with serum low concentrations of antioxidants (Murr et al., 2009). Therefore, neopterin may also represent an indirect sensitive indicator of oxidative stress during immune activation (Murr et al., 1999). In line with this, high neopterin concentrations were shown to indicate increased production of ROS in cancer patients (Murr et al., 1999; Hronek et al., 2000). Furthermore, tryptophan breakdown and neopterin formation served as readout to monitor and predict survival in cardiovascular diseases (Grammer et al., 2009; Pedersen et al., 2011).

Neopterin itself seems to accompany not only oxidative stress, but has also the ability to interact with ROS. Chemiluminescence experiments revealed that neopterin increases effects of reactive compounds such as H_2_O_2_, OCl^-^, chloramine, and ONOO^-^, whereas is sister compound 7,8-dihydroneopterin can act as a scavenger (Weiss et al., 1993). Cirillo et al. (2006) and Hofmann et al. (1992) reported that neopterin is able to induce the translocation of NF-κB in human endothelial cells and vascular smooth muscle cells.

**FIGURE 3:**
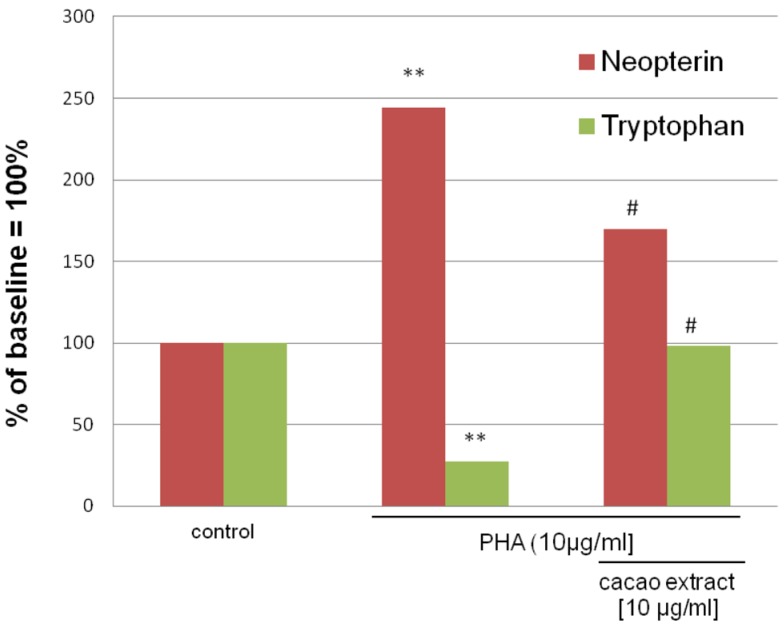
**Mean neopterin and tryptophan concentrations in mitogen (PHA)-stimulated freshly isolated peripheral blood mononuclear cells (PBMCs), either treated with 10 μg/ml aqueous cacao extracts or left untreated, compared to unstimulated controls.** The results are shown in % of the baseline in the supernatants of unstimulated PBMC. Experiments were run in duplicates and in four independent approaches [**p < 0.05 compared to unstimulated cells; #*p* < 0.05 compared to stimulated cells; adapted from Jenny et al. (2009)].

Aqueous extracts of cacao were found to significantly suppress tryptophan breakdown and neopterin formation in a dose-dependent manner *in vitro* (Jenny et al., 2009). In the experiments, neopterin formation was induced in human peripheral blood mononuclear cells (PBMCs) by the mitogen phythemaglutinin (PHA), treatment with cacao extract at non-toxic concentrations was able to inhibit tryptophan breakdown completely such that tryptophan concentration in the culture media returned to baseline (**Figure 3**). Furthermore, the formation of neopterin and IFN-γ was significantly suppressed. This result indicates potential anti-inflammatory and cell protective properties of cacao. The extracts of several plants and herbs used in traditional folk medicine like *Crinum latifolium*, *Camellia sinensis* (Zvetkova et al., 2001), or *Hypericum perforatum* (Winkler et al., 2004) had similar activities to suppress Th1-type immune activation pathways like production of IFN-γ and neopterin in PBMC system. These findings agree with the properties of other antioxidant components such as vitamins or antioxidant drugs, which also have suppressive effects on activated immune cells (Jenny et al., 2011).

Of note, treatment of myelomonocytic THP-1 cells with cacao extracts did not result in a reduction of lipopolysaccharide (LPS)-induced neopterin formation or tryptophan breakdown. Thus, the suppressive effects of cacao extracts are suggested to be exerted rather on T-cells signaling and IFN-γ production than on isolated macrophages (Jenny et al., 2009).

## CACAO AND MOOD DISORDERS

Patients with chronic diseases like infections, autoimmune diseases or cancer, have an increased risk for the development of mood disorders. Reasons can be either the poor or impaired future perspectives, but also the metabolic changes that are associated with diseases (Capuron et al., 2002; Widner et al., 2002). Depressive symptoms can also develop as side effects in patients under treatment with pro-inflammatory cytokines as interleukins, interferons. All these conditions indicate a close connection of cytokine-induced biochemical changes and development of neuropsychiatric symptoms (Capuron et al., 2003; Dantzer and Kelley, 2007).

Dysfunctions of neurotransmitter, hormone and vitamin synthesis and metabolism are involved in the pathogenesis of depressive disorders. For example, reduced concentration of 5-hydroxyindoleacetic acid, the main catabolite of serotonin, indicates insufficient serotonin availability. Selective serotonin-reuptake inhibitors (SSRIs) can counteract low levels of neurotransmitters and are frequently used in the treatment of anxiety and mood disorders (DeVane, 1998), although not always effective.

The neurotransmitter serotonin, dopamine, and norepinephrine share similarities in their synthesis, e.g., all of them are depending on enzymes that require BH_4_ as cofactor. A close relationship between high neopterin levels and BH_4_ biochemistry can be constructed with the critical involvement of BH_4_ in the biosynthesis of biogenic amines like serotonin and several adrenergic/dopaminergic neurotransmitters. This relationship is further supported by associated occurrences of these immunological markers and neuropsychiatric symptoms (Neurauter et al., 2008), e.g., patient suffering from seasonal affective disorders were shown to have low plasma levels of tryptophan and biopterin (a BH_4_ product), but elevated neopterin concentrations (Hoekstra et al., 2003).

During inflammation, the activated tryptophan breakdown leads to insufficient serotonin biosynthesis, which can increase the development of mood disturbances and depression and furthermore may influence cognitive functions (Neurauter et al., 2008). Also, tryptophan breakdown products such as 3-hydroxyanthranillic acid and quinolinic acid may negatively affect neurological functions, while other metabolites such as kynurenic acid can be neuroprotective (Heyes et al., 1992; Sas et al., 2007; Klein et al., 2013). Furthermore, an excess in ROS, which is produced during immune response, can interrupt the oxidation sensitive pathways like serotonin production.

A variety of clinical studies and experimental data have shown that tryptophan levels are low and neopterin levels are increased in patients with depression. These results support the hypothesis that the immune response plays a major role in development of depression (Maes et al., 1994; Murr et al., 2000).

Depression also develops in most of the IFN-α and/or IL-2 cytokine therapies (Capuron et al., 2003). During these therapies, a significant decrease of tryptophan concentrations can be monitored and also the ratio of tryptophan to large neutral amino acids like tyrosine, phenylalanine, leucine, and isoleucine is affected. These amino acids are known to correlate with depressive symptoms (Capuron et al., 2002, 2011). Thus, depressive disorders might represent a result of the tryptophan depletion which affects downstream pathways at multiple levels.

Due to its strong anti-inflammatory effects, cacao can influence on IDO activity as it has been shown *in vitro*, were treatment of mitogen-stimulated PBMC with cacao extracts resulted in an inhibition of tryptophan breakdown and neopterin formation in a dose-dependent manner (Jenny et al., 2009). Similar effects could possibly occur also *in vivo* and contribute to a mood-enhancing effect by improving tryptophan availability for serotonin synthesis.

The capacity to improve mood, lift spirits and make people feel good has been described for cocoa, cacao, and chocolate products. Chocolate was reported to have an antidepressant benefit on humans, to be a kind of self-medication in an atypical or a seasonal depression and to have a positive impact on brain neurotransmitters. Chocolate craving has some addictive features, and some psychoactive ingredients have been identified, like the biogenic stimulant amines caffeine, theobromine, tyramine, and phenylethylamine. However, the concentrations of these compounds in cacao are not that high and this effect cannot be attributed to them alone (Parker et al., 2006). Nevertheless, cacao can be a kind of indirect oral tryptophan supplementation by inhibiting IDO activity.

**FIGURE 4:**
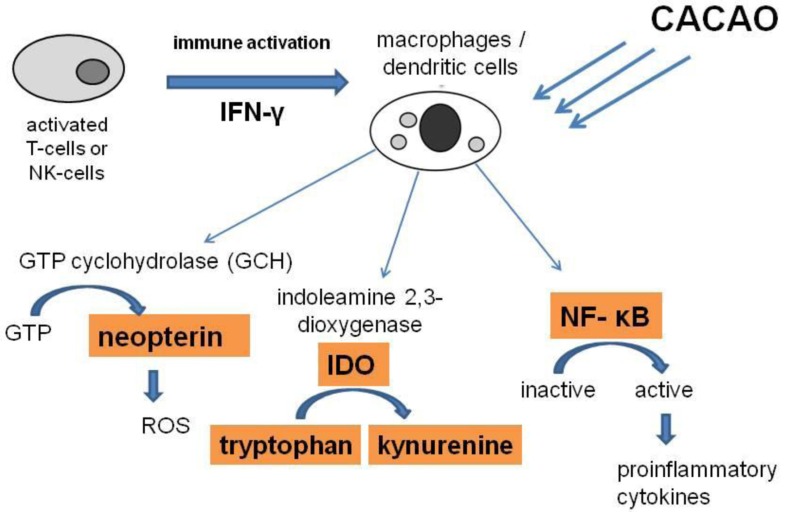
**During Th1-type immune response, activated T-cells and natural killer (NK) cells release large amounts of the inflammatory cytokine interferon-γ (IFN-γ), which triggers various immunoregulatory and anti-proliferative activities in target cells like macrophages (MΦ) and dendritic cells (DC).** Indoleamine 2,3-dioxygenase (IDO) converts tryptophan into kynurenine. In parallel GTP-cyclohydrolase I (GCH) is induced to produce neopterin out of GTP. Neopterin plays a major role in the release of reactive oxygen species (ROS) in human macrophages. When oxidative stress is rising to higher levels, transcription factor nuclear factor-κB (NF-κB) becomes activated and induces the pro-inflammatory signaling pathways. Cacao, acting as an antioxidant, can influence and counteract against these cascades.

Several cocoa constituents have been reported to reach the gastrointestinal tract. It was shown that, e.g., most ingested procyanidins arrive intact in the small intestine and are available for absorption or metabolism (Rios et al., 2002). Flavan-3-ol metabolites could be detected in plasma and serum after consumption of cocoa beverages (Mullen et al., 2009). These substances can accumulate at relatively high concentrations in the gastrointestinal tract, and an interference with tryptophan metabolism via the inhibition of IDO and consequently the disposability of serotonin (**Figure 4**) might be proposed. The gut is not only rich in the number of lymphocytes, about 95% of the human serotonin is synthesized and stored in the gastrointestinal tract, where it acts as a paracrine messenger to modulate sensation, secretion, and motility (Gershon and Tack, 2007). Another influence on neurological functions was reported by Bisson et al. (2008) who reported a decrease of cognitive impairments, which occur normally in aged rats, after long-term treatment with cocoa extracts.

## CONCLUSION

Diseases accompanied by chronic inflammation are frequently associated with depressive symptoms and mood disorders. Cellular immune activation is characterized by accelerated tryptophan breakdown and increased neopterin formation, which both can be used as sensitive biomarkers *in vitro* and *in vivo*. Tryptophan is degraded via IDO to kynurenine, which can further be metabolized to neuroactive products. Additionally, tryptophan is utilized for serotonin synthesis.

Cacao has the ability to decrease tryptophan breakdown, neopterin formation, and the concentration of other inflammatory markers under certain experimental conditions. Beneficial effects of cacao/cocoa could be expected if the breakdown of tryptophan can be rescued also *in vivo*, to guarantee enough tryptophan for the serotonin production. Antioxidant constituents could further decrease oxidative stress and interfere with redox-regulated pathways.

Nevertheless, it should keep in mind that most of the reported studies on cocoa/cacao compounds and extracts were performed *in vitro* only, and a direct extrapolation to the *in vivo* is not possible. Furthermore, although also processed cocoa products such as chocolate or cacao still contain a number of bioactive substances, these processed products are usually ingested within a complex food matrix and health effects are not always identical with natural cocoa extracts.

## Conflict of Interest Statement

The authors declare that the research was conducted in the absence of any commercial or financial relationships that could be construed as a potential conflict of interest.
